# Characterization and phylogenetic analysis of the complete mitochondrial genome sequence of *Photinia serratifolia*

**DOI:** 10.1038/s41598-022-24327-x

**Published:** 2023-01-14

**Authors:** Ying Wang, Shengjia Chen, Jiajun Chen, Chaojie Chen, Xiaojian Lin, He Peng, Qian Zhao, Xingya Wang

**Affiliations:** School of Pharmacy, Zhejiang Chinese Medical University, Hangzhou, 311400 Zhejiang People’s Republic of China

**Keywords:** Genetics, Genome, Mitochondrial genome

## Abstract

Plant mitochondrial genomes (mitogenomes) are a valuable source of genetic information for a better understanding of phylogenetic relationships. However, no mitogenome of any species in the genus of *Photinia* has been reported. In this study, using NGS sequencing, we reported the mitogenome assembly and annotation of *Photinia serratifolia*, which is 473,579 bp in length, contains 38 protein-coding genes, 23 tRNAs, and 6 rRNAs, with 61 genes have no introns. The *rps2* and *rps11* genes are missing in the *P. serratifolia* mitogenome. Although there are more editing sites (488) in the *P. serratifolia* mitogenome than in most angiosperms, fewer editing types were found in the *P. serratifolia* mitogenome, showing a clear bias in RNA-editing. Phylogenetic analysis based on the mitogenomes of *P. serratifolia* and 8 other taxa of the Rosaceae family reflected the exact evolutionary and taxonomic status of *P. serratifolia*. However, Ka/Ks analysis revealed that 72.69% of the protein-coding genes in the *P. serratifolia* mitogenome had undergone negative selections, reflecting the importance of those genes in the *P. serratifolia* mitogenome. Collectively, these results will provide valuable information for the evolution of *P. serratifolia* and provide insight into the evolutionary relationships within *Photinia* and the Rosaceae family.

## Introduction

The genus *Photinia* are evergreen plants that belongs to Rosaceae family, comprising approximately 60 species^1^. *Photinia* species are widely cultivated throughout the world and many are cultivated for gardening due to resistance to air pollution and other environmental stressors^2,3^. *P. serratifolia* (syn, *Photinia serrulata*), commonly called as Taiwanese photinia or Chinese hawthorn, is widely cultivated in Southeast Asia, native to China, India, Japan, Indonesia, and the Philippines. *P. serratifolia* not only has high ornamental value, but also has medicinal value, which is a well-known herb in traditional Chinese medicine (TCM) for the treatment of rheumatism, nephropathy, and spermatorrhea^4^. Its tender leaves are used as edible vegetables in the south of China, and the matured leaves are used for the treatment of the above diseases^4^. It has been reported that the leaves of *P. serratifolia* contain essential oils, triterpenoids, flavonoids, polyphenols, and other bioactive compounds, which have been demonstrated to have antioxidant and anticancer activities in vitro^4,5^.

The boundaries of some species have not been clearly defined in Rosaceae family due to similar morphological features exhibiting among Rosaceae species^1^. Compared with morphological identification, DNA sequences can produce more accurate phylogenetic relationships^1,6^. The phylogenetic study has greatly advanced the studies of taxonomic reclassification with the rapid advances in DNA technologies^7,8^. Both chloroplast genomes and mitogenomes have been widely used in phylogenetic studies among species^6,9^. To date, more than 5000 plant chloroplast genomes have been sequenced, but only about 424 plant mitogenome sequences are available (https://www.ncbi.nlm.nih.gov/genome/organelle/, 2/12/2022). Mitochondria are the main organelles involved in energy metabolism in eukaryotic cells^10,11^. In plant, mitochondria play an important role in plant productivity, development, and various biochemical processes^12–14^. According to endosymbiotic theory, plant mitochondria are believed to have descended from free-living bacteria-independent microorganisms, which explains the presence of their genomes^15,16^.

Plants have about 100–10,000 times larger and more structurally complex mitogenomes than animals^17–21^. During evolution, the plant mitogenome underwent dramatic changes in, for example, the gene order, genome structure, and migration of sequences from other organelles^17–19^. The mitogenomes of plants demonstrate significant variations in size and structure organization^20^. For example, the genome size can vary from 66 kb of *Viscum scurruloideum*^21^ to 11.3 Mb of *Silene conia*^22^. The number of protein-coding genes varies from 33 of *Arabidopsis thaliana*^23^ to 74 of *Vitis vinifera*^24^. The number of tRNA genes varies from 3 of *Rosa chinensis*^25^ to 31 of *V. vinifera*^24^. In addition, the plant mitogenome has numerous repetitive sequences and multiple RNA editing modifications^26^. In contrast to the conserved structure of plant chloroplast genomes, the variations in mitogenomes are not only between plant species but also can be within the same species^12,17,22^. For these reasons, mitogenomes have been used as a valuable source of genetic information and for the investigation of essential cellular processes in many phylogenetic studies. However, the characteristics of plant mitogenomes (bigger size, more structural complexity, and low conservation across species) make plant mitogenome assembly difficult^13,14^. Fortunately, advancements in long-read sequencing, such as PacBio and Oxford Nanopore, have made organelle genome sequencing easier and faster.

Recently, the complete chloroplast genome sequences of *Photinia* × *fraseri*, *Photinia davidsoniae*, and *Photinia glabra* have been sequenced and published^1,27,28^. However, at present, no mitogenome of any species in *Photinia* has been reported. Therefore, in this study, we constructed the complete mitogenome of *P. serratifolia* based on Oxford Nanopore and Illumina data, performed a phylogenetic analysis, and compared the complete mitogenomes of *P. serratifolia* and related family. Our results will help better understand the features of the *P. serratifolia* mitogenome and lay the foundation for identifying further evolutionary relationships within Rosaceae.

## Results and discussion

### Sequencing and genome structure of the complete mitogenome of *P. serratifolia*

**Figure 1:**
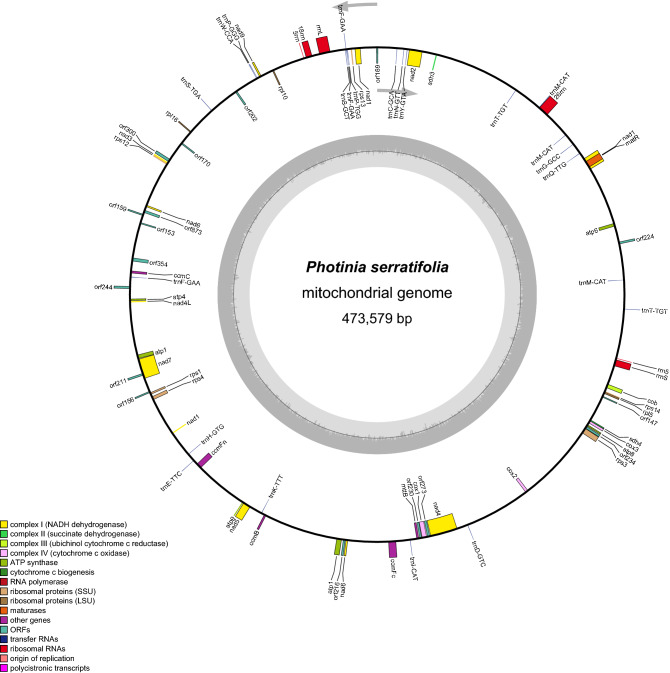
The circular map of *P. serratifolia* mitogenome. Gene map showing 68 annotated genes of different functional groups.

The total DNA of *P. Serratifolia* was sequenced, and the raw data had been prepared for assembly, resulting in 115.88 G Nanopore PromethION sequencing data with an average read length of 23,654 bp (61–26,706 bp) and 34.3 G Illumina sequencing data (Supplementary Table S1). We then assembled the complete mitogenome of *P. serratifolia* in a circular contig of 473,579 bp (Fig. 1), which has been deposited in the NCBI Genome Database (GenBank accession number: MZ153172). The mitogenomes of 19 species were selected for analysis in this study (Supplementary Table S2). It is well known that the plant mitogenome greatly varies in size, from 66 kb in *V. scurruloideum*^21^ to 11.3 Mb in *S. conica*^22^. As shown in Supplementary Table S2, the relatively medium size of the *P. serratifolia* mitogenome was smaller than that of *Zea mays* (680,603 bp) and *Oryza sativa* (490,520 bp). However, the mitogenome of *P. serratifolia* was slightly larger than *Pyrus betulifolia* (469,928 bp), *Rhaphiolepis bibas* (434,980 bp), and *Malus hupehensis* (422, 555 bp), and significantly larger than that of *Sorbus aucuparia* (384,977 bp) and *Sorbus torminalis* (386,758 bp). These results suggest that *P. serratifolia* may be identified as a species with a larger mitogenome in the Rosaceae family.

The nucleotide composition of the whole mitogenome is A: 27.6%, T: 27.2%, C: 22.7%, and G: 22.5%, and the overall GC content was 45.2% (Supplementary Table S2), which is consistent with that of most of the species of Rosaceae family we compared (*M. hupehensis*: 45.21%; *Malus domestica*: 45.4%; *Prunus avium*: 45.62%; *P. betulifolia*: 45.28%; *S. aucuparia*: 45.39%; *S. torminalis*: 45.31%) and other angiosperms (*Ziziphus jujuba*: 45.27%; *A. thaliana*: 44.79%; *Glycine max*: 45.03%), but smaller than some gymnosperm, such as *Ginkgo biloba*: 50.36%.

### Gene contents of the mitogenome of *P. serratifolia*

Although the genome size of plant mitochondrial greatly varied, the number of mitochondrial genes is relatively conserved in the land plant lineage, with 60–80 known genes found in different terrestrial plant species^29^. In the *P. serratifolia* mitogenome, 67 genes (38 protein-coding genes, 23 tRNA genes, and 6 rRNA genes) were annotated (Supplementary Table S2). The functional categorization and physical locations of the annotated genes were shown in Fig. 1. The 38 encoded proteins (*nad6* and *atp1* have two copies) could be divided into 11 classes: ATP synthase (6), cytochrome C biogenesis (4), ubiquinol cytochrome c reductase (1), cytochrome C oxidase (3), maturases (1), transport membrane protein (1), NADH dehydrogenase (10), ribosomal proteins (large subunit (LSU); 3), ribosomal proteins (small subunit (SSU); 6), succinate dehydrogenase (2), and ribonuclease (1) (Supplementary Table S3).

**Figure 2:**
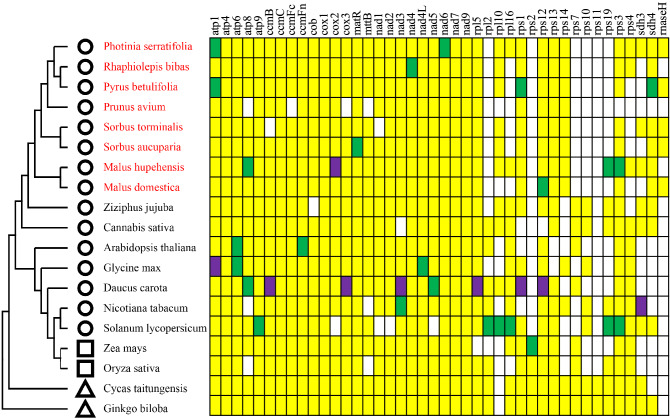
Distribution of protein-coding genes in plant mitogenomes. Yellow, green, and purple boxes indicate that one, two, and three copies exist in the plant mitogenome, respectively. White boxes indicate that the gene is missing in the plant mitogenome. The circles, squares, and triangles represent dicots, monocots, and gymnosperms, respectively. Besides, the red-colored plant names are species from the Rosaceae family.

Although comparative analyses of mitogenomes have shown that the sequences of protein-coding genes are highly conserved in plants, variations among plant mitogenomes characterized so far have mainly been reported in the ribosomal proteins^30,31^. In addition, the gene components cytochrome c biogenesis gene has also been reported to be different among the plant mitogenomes^32^. Interestingly, consistent with previous mitogenome studies of Rosaceae^33^, most *rps* genes (*rps2*, *rps7*, *rps10*, *rps11*, *rps19*) were missing in the mitogenome of *P. serratifolia* (Fig. 2). The functions of missing ribosomal genes may be replaced by nuclear genes, which may be related to the rapid radiation evolution of Rosaceae plants^34^. Although there was no significant variation of the composition of cytochrome C synthase gene among other species of the Rosaceae family in our study, the length of *ccmFc*, *ccmFn*, *cob*, *cox1*, *cox2*, and *cox3*, in the mitogenome of *P. serratifolia*, *R. bibas*, and *M. hupehensis*, were 797–2271 bp, which was significantly higher than that of other species (212–587 bp) of the family.

Other than ribosomal proteins, the major variations characterized among plant mitogenomes, even in the same genus, are in the tRNA gene contents^30^. The *P. serratifolia* mitochondria had 23 tRNAs (Supplementary Table S3). The average length of these tRNAs was 71–87 bp, with a total length of 1725 bp (Supplementary Table S3). The number of tRNAs in the *P. serratifolia* mitogenome was more than that in other species of the Rosaceae family, such as *R. bibas* (22), *M. domestica* (20), *P. avium* (16), and *S. torminalis* (18) (Supplementary Table S2). This may be because some tRNAs in the *P. serratifolia* mitogenome have multiple copies. For example, trnfM-CAT and trnF-GAA have two copies. The function of the missing mitochondrial tRNAs may be replaced by chloroplast-derived tRNAs in species with less mitochondrial tRNAs^34^. Moreover, consistent with the previous report^35^, we found that protein-coding genes of the *P. serratifolia* were not increased along with the increase of tRNAs.

Furthermore, we found that 61 out of the 67 mitochondrial genes have no introns, accounting for 92.54% of the total. Our result is consistent with the general consensus that 63.2% to 100% of mitochondrial genes in most plants have no introns^17,18^. However, six mitochondrial genes (*ccmFC*, *nad5*, *nad1*, *nad2*, *nad4*, and *nad7*) are found to contain one or more introns of the *P. serratifolia* (Supplementary Table S3).

### Repeat sequences analysis

**Figure 3:**
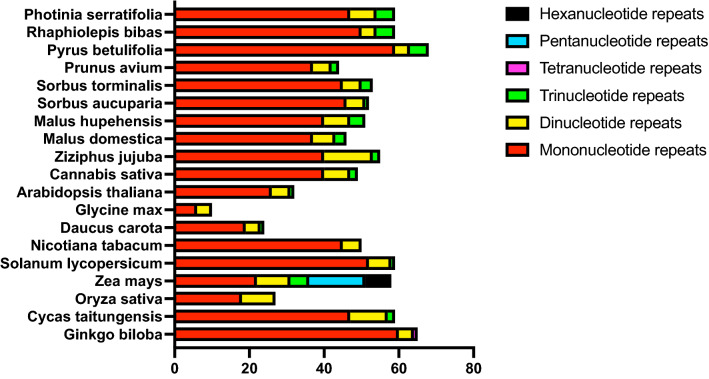
The SSRs composition in plant mitogenomes.

SSRs, or microsatellites, are DNA stretches consisting of short, tandem units of sequence repetitions of 1–6 base pairs in length^36^. In the current study, we identified 59 SSRs in the *P. serratifolia* mitogenome. The proportions of different repeat units were shown in Fig. 3. Consistent with all observed species, mononucleotide repeats were the most abundant SSR type in *P. serratifolia*, constituting 79.67% (47 repeats) of all identified SSRs. In addition, there were 7 SSRs (11.86%) and 5 SSRs (8.47%) in di-, trinucleotide repeats, respectively. However, there were no tetra-, penta-, and hexa-repeats identified in *P. serratifolia* mitogenome. The mononucleotide repeats of A/T motifs (a total of 41 repeats) were the most recurrent motifs, representing 69.49% of all identified SSRs (Supplementary Table S4). According to the trend that the distribution pattern of microsatellites is consistent with their phylogenetic status in plants^37^, the SSR composition of *P. serratifolia* was similar to its most closely related species, such as *R. bibas* and *P. betulifolia* (Fig. 3).

In addition, 72 non-tandem repeats, with 50 bp or more in length, were detected in the *P. serratifolia* mitogenome (Supplementary Table S5). The repetitive sequence in the *P. serratifolia* mitogenome was 51.05 kb, accounting for 10.78% of the mitogenome. The proportion of repeats is higher than that in *Garcinia mangostana* (5.8%)^38^ and *Prunus salicina* (7.22%)^39^, but lower than that in *Nicotiana tabacum* (13%)^40^ and *Daucus carota* (16%)^41^. The different proportions of repeats may be because the mitochondria of *G. mangostana* and *P. salicina* are mainly short repeating units, whereas those of *P. serratifolia* and *D. carota* are mainly longer repeating units^41^.

**Figure 4:**
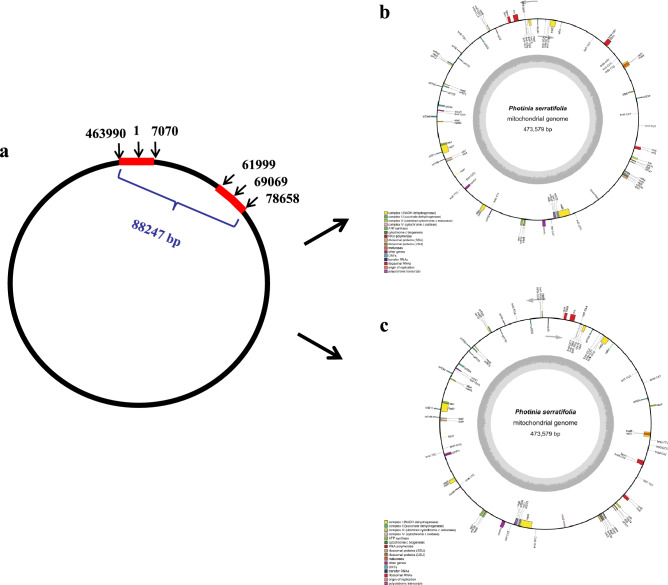
The distribution of the pair of long repeats and the possible configurations generated from inversions of these long repeats. a: The distribution of the pair of long repeats (16660 bp) (red bars) in the *P. serratifolia* mitogenome. b and c: Two possible configurations generated from inversions of these long repeats. b is the master conformations, which is same as Fig. 1 shown. c is an alternative configuration of mitogenomes of *P. serratifolia.*

**Figure 5:**
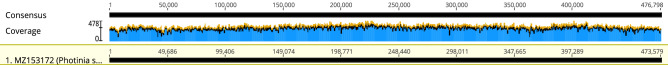
Depth and coverage of the assembled mitogenome using sequencing long-reads. The abscissa shows the genomic positions, and the ordinate shows the depth of mapped raw reads.

For example, we found one pair long repeat (16,660 bp), one copy at the starting and ending positions of the genome (463990-473579-1-7070), another at 61,999–78,658 bp (Fig. 4a), and 16 pair medium sized repeats between 120 and 920 bp in the *P. serratifolia* mitogenome (Supplementary Table S5). The distribution of repeat is consistent with many plant mitogenomes that have one or more pairs of large repeats^38,42,43^. Some reports showed that larger and medium-sized repeats can act as sites for inter- or intramolecular recombination, leading to multiple alternative arrangements or isoforms^42,43^. Although the frequency of recombination events was low, all these sequencing reads were aligned to the *P. serratifolia* mitogenome for the detection of potential alternative isoforms. As a benefit of Nanopore PromethION sequencing, these ultra-long reads of *P. serratifolia*, with an average read length of 23,654 bp, is longer than these identified repeats. Therefore, the long reads can cover identified repeats with high probability. As shown in Fig. 5, the sequencing reads coverage of these repeats is similar to those of other non-repetitive sequences, which implies no branching nodes in each repeat. Therefore, *P. serratifolia* mitochondrial master genome assembly can be represented in the circular form, as previously reported in plant mitogenomes^20,38,44^. However, there is a total length of 88,247 bp between the two copies of the long repeats (Fig. 4a), which may give rise to an alternative configuration of mitogenomes via inversions of these long repeats in master conformation (Fig. 4b,c).

### The prediction of RNA editing in the *P. serratifolia* mitogenome

**Figure 6:**
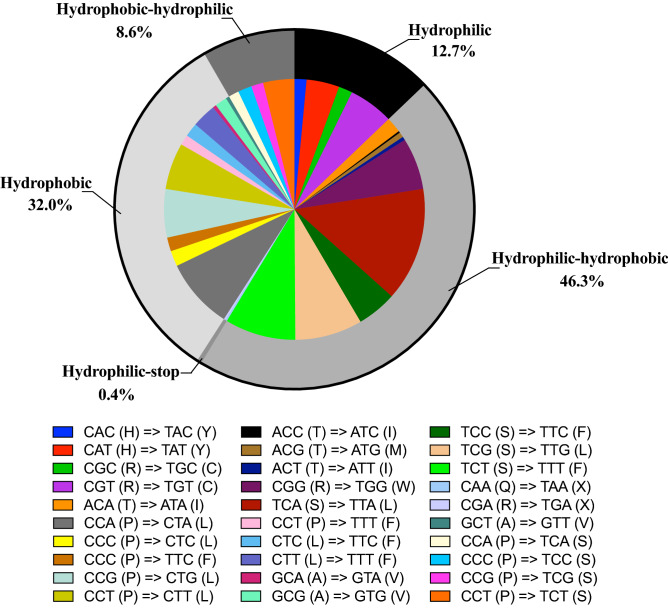
Prediction of RNA editing sites in the *P. serratifolia* mitogenome.

The number of RNA-editing sites varies in different species and is usually frequent in angiosperm and gymnosperm mitochondria^45^. We predicted 488 RNA-editing sites within the 33 protein-coding genes (Fig. 6) in the *P. serratifolia* mitogenome, which was similar to those in *A. thaliana* (441 sites)^15^, *Eucalyptus grandis* (470 sites)^46^, and *Citrullus lanatus* (463 sites)^47^ and less than those in gymnosperms that have larger mitogenomes, such as *Taxus cuspidata* (974 sites), *Pinus taeda* (1179 sites), *Cycas revoluta* (1206 sites), and *G. biloba* (1306 sites)^48^. However, whether the number of RNA-editing sites is positively correlated with the size of the mitogenome requires further research.

The selection of mitochondrial RNA-editing sites in *P. serratifolia* shows a high degree of compositional bias. As shown in Fig. 6, all RNA-editing sites are the C-T editing type, which is consistent with the fact that C-T is the most common editing type found in plant mitogenomes^49,50^. Inconsistent with previous studies^50^, more than half (313 sites, 64.14%) of the mitochondrial RNA editing occurred at the second codon position in *P. serratifolia* (Fig. 6), followed by that at the first codon position (161 sites; 32.99%) (Fig. 6). However, no editing site was found at the third position of triplet codons, consistent with the fact that RNA-editing sites at this position were rare in plant mitogenomes^48,49^.

Although the *P. serratifolia* mitogenome has more RNA-editing sites, and the vast majority of RNA editing occurs at the first or second position of codons, there were only 30 codon transfer types, corresponding to 14 amino acid transfer types, suggesting a consolidated biological function. The types of transfer are comparable to those of most gymnosperms (30–40 codons; around 20 amino acids)^48,50^ but less than those of monocotyledonous and dicotyledonous plants (50–60 codons; around 30 amino acids)^46,47,49^. Among the 30 codon transfer types, TCA =  > TTA was the most common type, with 68 sites. A leucine tendency after RNA editing, supported by the fact that 44.88% (219 sites) of the edits were converted to leucine, was found in the amino acids of predicted editing codons. After RNA editing, 32.0% of the amino acids remained hydrophobic. However, 46.3% of the amino acids were predicted to change from hydrophilic to hydrophobic, while 8.6% were predicted to change from hydrophobic to hydrophilic. Overall, our study suggests that the *P. serratifolia* mitogenome has more RNA-editing sites but fewer editing types.

**Figure 7:**
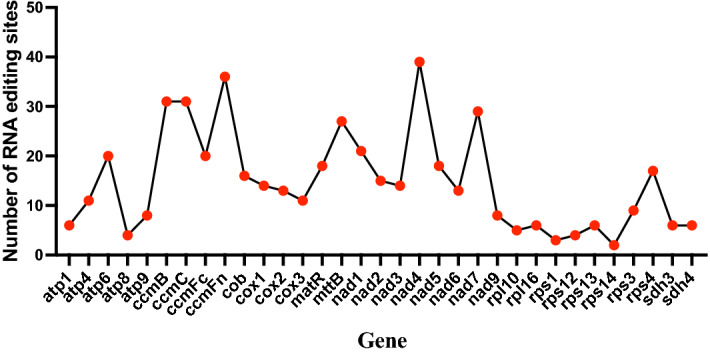
The distribution of RNA-editing sites in the *P. serratifolia* mitochondrial protein-coding genes.

It has been well established that RNA editing is an epitranscriptomic mechanism that modifies primary RNAs, and is widespread in plants organelles^51^, Fig. 7 shows the total number of editing sites of all of the 33 protein-coding genes. Although the pattern changes of RNA editing extent varies between different plant species^52^, similar to most angiosperms^50^, ribosomal proteins (except *rps4*) and ATPase subunits (except *atp6*) had a relatively small number of RNA-editing-derived substitutions (2–11 sites), while the transcripts of NADH dehydrogenase subunits and cytochrome c biogenesis genes were significantly edited (13–39 sites; Fig. 7) in the *P. serratifolia* mitogenome. Consistent with the previous report, such as *Phaseolus vulgaris*^26^ and *Suaeda glauca*^53^, *nad4* (36 sites), *ccmFn* (39 sites), and *ccmB* (31 sites) had the highest total number of RNA-editing sites predicted in the *P. serratifolia* mitogenome (Fig. 7). This supports the essential role of editing sites in the proper functioning of mitochondrially encoded proteins.

### Codon usage and Ka/Ks analysis

**Figure 8:**
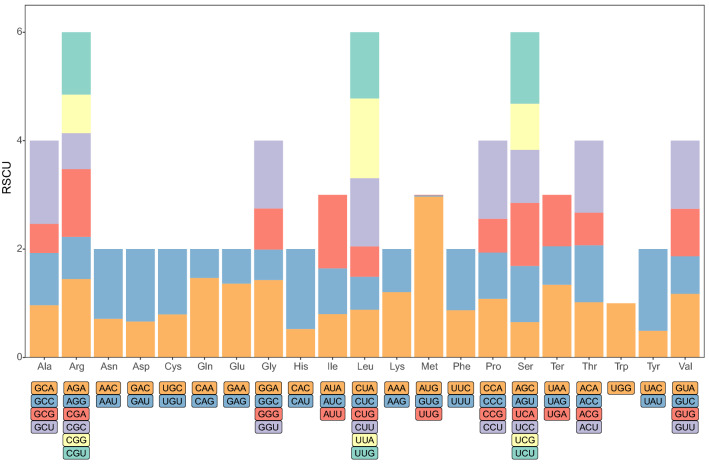
Relative synonymous codon usage in the *P. serratifolia* mitogenome.

As shown in Supplementary Table S3, in the *P. serratifolia* mitogenome, ATG was used as the starting codon by almost all the protein-coding genes, while *mttB* starts with TTG, *rpl16* and *rps4* start with GTA as the start codon. Three types of stop codons, TAA, TGA, and TAG, were found in the *P. serratifolia* mitogenome which had utilization rates of 44.7%, 31.6%, and 23.7%, respectively (Supplementary Table S3). The relative synonymous codon usage (RSCU) value for *P. serratifolia* for the third codon position is shown in Fig. 8. Consistent with most of the currently studied mitogenomes^10,53,54^, the use of both two- and four-fold degenerate codons was biased toward the use of codons abundant in A or T. In *P. serratifolia*, 14,333 amino acids were encoded. The most frequently used amino acids were Leu (7.1%), Arg (6.3%), and Ser (6.1%), and the least common amino acids were Trp (1.4%) and Met (1%) (Fig. 8).

**Figure 9:**
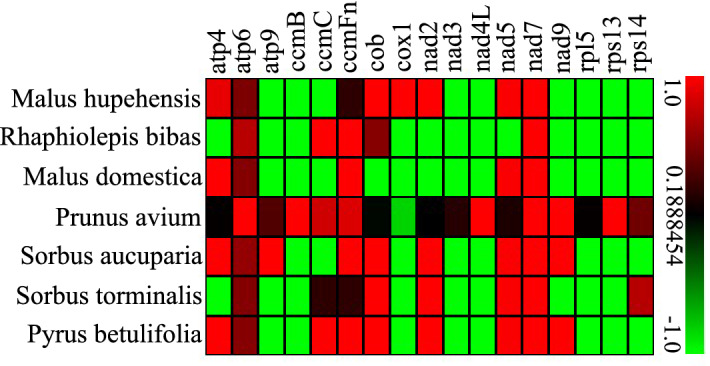
The Ka/Ks values of 17 protein-coding genes of *P. serratifolia* versus 7 species. The color in each box represents the Ka/Ks value.

In genetics, the Ka and Ks substitution ratio (Ka/Ks) is useful for inferring the direction and magnitude of natural selection across diverged species^55^. A Ka/Ks ratio < 1 implies negative selection, while a ratio of > 1 implies positive selection (driving change) and a ratio of exactly 1 indicates neutral selection. To evaluate selective pressures during the evolutionary dynamics of protein-coding genes among closely related species, the Ka/Ks ratio of 17 single copy PCGs among *P. serratifolia* and 7 Rosaceae species mitogenomes was calculated. As shown in Fig. 9, there was no substitution in most mitochondrial genes, such as *rpl5*, *rps13*, *rps14*, *nad3*, *nad4L*, *atp9*, *ccmB*, and *cox1*, among *P. serratifolia* and other seven species in Rosaceae. More frequency changes were found in *atp* genes among species.

In 21 cases (Fig. 9), Ka/Ks values of *P. serratifolia* gene-specific substitution rates were higher than 1. This result suggests a positive selection during the evolution of *P. serratifolia* as compared with 7 other species^55,56^ Among these cases, the Ka/Ks values of the *nad* gene-specific substitution rates of *P. serratifolia* were higher, with Ka/Ks values of 7 *nad7* genes and 4 *nad3* > 1, suggesting large variation and positive selection during *nad* gene evolution among Rosaceae^55^. However, most genes had undergone negative selection pressures during evolution, supported by the fact that the Ka/Ks values of 86 proteins-coding genes, accounting for 72.69% of the proteins-coding genes, were less than 1 compared to the other plant species. Taken together, these results suggest that mitochondrial genes are highly conserved during the evolutionary process in Rosaceae plants.

### Phylogenetic analyses

**Figure 10:**
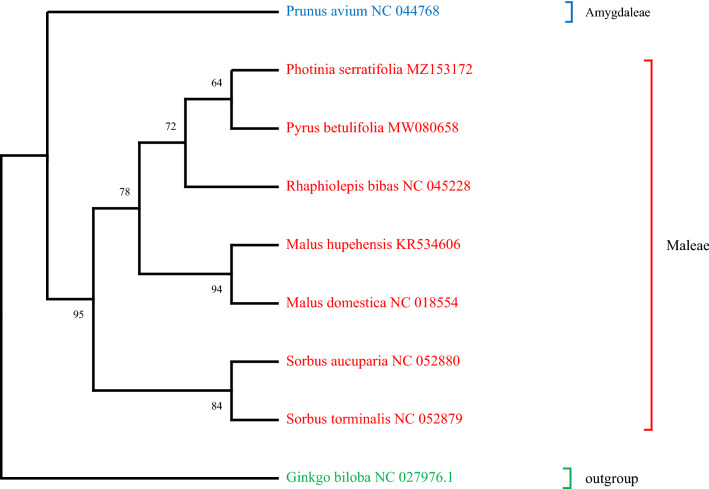
The phylogenetic relationships of *P. serratifolia* with other 8 plant species using the ML analysis. The bootstrapping values are listed in each node. The number after the species name is the GenBank accession number. Colors indicate the groups that the specific species belongs.

To detect the evolutionary status of the *P. serratifolia* mitogenome, a phylogenetic analysis was performed on *P. serratifolia*, together with 8 other species. Phylogenetic relationships (Fig. 10) were analyzed using the concatenated dataset by 17 PCGs through ML phylogenetic analysis. The abbreviations and accession numbers of the mitogenomes investigated in this study are listed in Supplementary Table S2. As shown in Fig. 10, as outgroups, the *G. biloba*, which belongs to gymnosperm, was distinct from the other angiosperms. Moreover, the taxa of the 7 Rosaceae species were well clustered. Among the Rosaceae cluster, *P. avium*, which belongs to Amygdaleae subfamily, was distinct from the other 7 species of Maleae subfamily, which also supports the classification of Amygdaleae and Maleae subfamily^57,58^. Meanwhile, these species in the same genus were clustered together, such as *S. aucuparia* and *S. torminalis*, *M. hupehensis* and *M. domestica*, which is consistent with previous reports based on morphological and genetic data^57–59^.

In addition, we also found that the clade united *P. serratifolia* with *P. betulifolia* (Fig. 10). The present phylogenetic analysis shows that *R. bibas* is sister to *P. serratifolia* + *P. betulifolia*, which is consistent with the previous report^60^. Our results also support the groupings (*Sorbus* + (*Malus* + (*Rhaphiolepis* + (*Photinia* + *Pyrus*)))), which have been partly supported in the previous study^61^. However, more accurate sequence and increased taxa sampling are necessary to further research the monophyly of these genus at the mitogenomes level. In general, the phylogenetic tree topology was in line with the evolutionary relationships among those species, indicating the consistency of traditional taxonomy with the molecular classification.

## Conclusions

In conclusion, the current study presented the first mitogenome assembly and annotation of *P. serratifolia* as well as the mitogenome in the genus *Photinia*. The mitogenome was 473,579 bp in length, containing 38 protein-coding genes, 23 transfer RNA genes, and 6 ribosomal RNA genes. Comparative analysis of gene structure, codon usage, repeat regions, and RNA-editing sites showed that *rps2* and *rps11* genes were missing, and a clear bias of RNA-editing sites is existing in the *P. serratifolia* mitogenome. Furthermore, the Ka/Ks analysis based on code substitution revealed that most of the coding genes had undergone negative selections, indicating the conservation of mitochondrial genes during the evolution. Moreover, Phylogenetic analysis based on the mitogenomes of *P. serratifolia* and 8 other taxa indicates consistency in molecular and taxonomic classification. These results will help in better understanding the features of the *P. serratifolia* mitogenome and lay the foundation for identifying further evolutionary relationships within Rosaceae.

## Materials and methods

### Mitochondrial DNA isolation and genome sequencing

The fresh young leaves of *P. serratifolia* were collected from City Forest Park in Fuyang of Hangzhou, Zhejiang province of China (119°58′8.4″ E, 30°4′20.28″ N) by Ying Wang and Xingya Wang, which were identified by Dr. Liang Xu of Zhejiang Academy of Forestry, Hangzhou, China. The voucher specimens were stored in the Herbarium of College of pharmaceutical sciences, Zhejiang Chinese Medical University, voucher No. ZCMU4C507. The collection of *P. serratifolia* was permitted by the City Forest Park. The use of plant leaves in this study complies with all local, national or international guidelines and legislation concerning research involving plants. Leaves were quickly frozen in liquid nitrogen and then stored at − 80 °C refrigerator prior to DNA isolation. High-quality genomic DNA was extracted using a modified cationic detergent cetyltrimethylammonium bromide (CTAB) method^62^. Sequencing was performed following the protocol for the BGISEQ-500 platform (BGI, Wuhan, China) and the library protocol for Nanopore PromethION sequencing (Pacific Biosciences, Menlo Park, CA, USA).

### Genome assembly

In this study, Raw data of second-generation sequencing were filtered using fastp version 0.20.0 software (https://github.com/OpenGene/fastp)^63^. The three-generation sequencing data of mitochondrial reads were error-corrected, trimmed, and de-novo-assembled using a Canu assembler (version 1.5) with default parameters^64^. Then, the contig sequence was obtained. The gene databases of plant mitochondria that published on the NCBI were compared using blast v2.6 (https://blast.ncbi.nlm.nih.gov/Blast.cgi), and contigs that matched with the mitochondrial genes as the seed sequence were selected. The original data were used to extend and circularize the contigs to obtain the ring-dominant structure (or secondary ring), and then, the assembly was polished using NextPolish 1.3.1 (https://github.com/Nextomics/NextPolish)^65^. The assembly results were calibrated using second- and third-generation data, and the parameters were set as rerun = 3 and -max_depth = 100. Then, the final assembly results were obtained.

### Genome annotation

The assembled *P. serratifolia* mitogenome was annotated using the GeSeq tool^66^ and MITOFY^47^. To confirm the annotated results, the assembled *P. serratifolia* mitogenome was also BLAST-searched against protein-coding genes and ribosomal RNA (rRNA) genes of available plant mitogenomes at the NCBI. Then, the sequence coordinates of the identified protein-coding genes (PCGs) were manually verified for start and stop codons. The annotations of transfer RNA (tRNA) genes were also confirmed by tRNAscan-SE 2.0^67^. Vi-ennarNA-2.4.14 was used to visualize the secondary structure of tRNA^68^. The possible RNA-editing sites in the PCGs of *P. serratifolia* were predicted using the online predictive RNA editor for plant mitochondrial genes (PREP-Mt) suite of servers (http://prep.unl.edu/)^69^. The codon frequencies were calculated using the Codon Usage tool in the Sequence Manipulation Suite (bioinformatics.org/sms2/codon_usage.html)^70^. The relative synonymous codon usage (RSCU) was calculated using the CAI Python package of Lee^71^. The physical circular map was drawn using the Organellar Genome DRAW (OGDraw) v1.2 program^72^. The final annotated mitogenome sequences of *P. serratifolia* have been deposited in the NCBI GenBank (accession no. MZ153172).

### Analysis of repeated sequence

The simple sequence and tandem repeats were detected in the *P. serratifolia* mitogenome. The MIcroSAtellite (MISA) identification tool Perl script was used to detect simple sequence repeats^73^. The repeats of mono-, di-, tri-, tetra-, penta-, and hexanucleotide bases with 10, 6, 5, 5, 5, and 5 repeat numbers, respectively, were identified. ROUSfinder was used for the identification of repeat elements^74^. Subsequently, in order to explore whether these identified repeats lead to the formation of multiple mitogenome isoforms, all these sequencing reads were aligned to the *P. serratifolia* mitogenome using Geneious Basic^75^. If there are a branching node in repeats, the coverage of reads at the branch will be halved^76^. In this way, the evidence of recombination that was mediated by repeat sequences could be observed directly.

### Phylogenetic tree construction and Ka/Ks analysis

The mitogenomes of previously reported mitochondrial assemblies were down-loaded from the NCBI Organelle Genome Resources Database for *R. bibas*, *P. betulifolia*, *P. avium*, *S. torminalis*, *S. aucuparia*, *M. hupehensis*, *M. domestica*, and *G. biloba* (designated as outgroups). These mitogenome sequences were selected due to they are clearly taxonomically classified. The conserved protein-coding genes from the mitogenomes of *P. serratifolia* and the above 8 species were identified and evaluated. Phylogenetic analyses were conducted using concatenated exon sequences from 17 conserved protein-coding genes (*atp4*, *atp6*, *atp9*, *ccmB*, *ccmC*, *ccmFn*, *cob*, *cox1*, *nad2*, *nad3*, *nad4L*, *nad5*, *nad7*, *nad9*, *rpl5*, *rps13*, and *rps14*), which were extracted and aligned using MAFFT v7.402 with default parameters^77^. ModelTest-NG v0.1.3 was used to determine the best-fit model^78^, and a maximum likelihood (ML) phylogenetic tree was generated using RAxMLv8.2.12 with the best-fit substitution model (GTR-GAMMA) at 1000 bootstrap replicates^79^. The synonymous (Ks) and nonsynonymous (Ka) substitution rates of the protein-coding genes in the *P. serratifolia* mitogenome were analyzed using the 7 species (*R. bibas*, *P. betulifolia*, *P. avium*, *S. torminalis*, *S. aucuparia*, *M. hupehensis*, and *M. domesticaand*). In this analysis, KaKs_Calculator (v2.0) with the MLWL model was used to calculate Ka/Ks^80^.

## Supplementary Information

Supplementary Information.

## Data Availability

The complete mitochondrial genome of *P. serratifolia* has been submitted to the NCBI database (https://www.ncbi.nlm.nih.gov/) under the accession number MZ153172.

## References

[1] Li J (2021). The complete chloroplast genome of Photinia davidsoniae: Molecular structures and comparative analysis. Mitochondr. DNA B.

[2] Mattei P (2017). Use of phytoremediated sediments dredged in maritime port as port as plant nursery growing media. J. Environ. Manage..

[3] Mori J (2018). Air pollution deposition on a roadside vegetation barrier in a Mediterranean environment: Combined effect of evergreen shrub species and planting density. Sci. Total Environ..

[4] Hou J (2007). Chemical composition, cytotoxic and antioxidant activity of the leaf essential oil of *Photinia serrulate*. Food Chem..

[5] Song Y (2007). Two new triterpenoids from *Photinia serrulata*. Molecules.

[6] Hiesel R, von Haeseler A, Brennicke A (1994). Plant mitochondrial nucleic acid sequences as a tool for phylogenetic analysis. Proc. Natl. Acad. Sci. U. S. A..

[7] Rouhan G, Gaudeul M (2021). Plant taxonomy: A historical perspective, current challenges, and perspectives. Methods. Mol. Biol..

[8] Yi J (2022). Novel gene rearrangement in the mitochondrial genome of *Anastatus fulloi* (Hymenoptera Chalcidoidea) and phylogenetic implications for Chalcidoidea. Sci. Rep..

[9] Clegg MT, Gaut BS, Learn GH, Morton BR (1994). Rates and patterns of chloroplast DNA evolution. Proc. Natl. Acad. Sci. U. S. A..

[10] Lü J (2022). The mitochondrial genome of *Grapsus albolineatus* (Decapoda: Brachyura: Grapsidae) and phylogenetic associations in Brachyura. Sci. Rep..

[11] Sureshan SC (2021). Complete mitochondrial genome sequencing of *Oxycarenus laetus* (Hemiptera: Lygaeidea) from two geographically distinct regions of India. Sci. Rep..

[12] Ogihara Y (2005). Structural dynamics of cereal mitochondrial genomes as revealed by complete nucleotide sequencing of the wheat mitochondrial genome. Nucleic Acids. Res..

[13] Wang X (2021). A complete sequence of mitochondrial genome of Neolamarckia cadamba and its use for systematic analysis. Sci. Rep..

[14] Cui H (2021). Comparative analysis of nuclear, chloroplast, and mitochondrial genomes of watermelon and melon provides evidence of gene transfer. Sci. Rep..

[15] Greiner S, Bock R (2013). Tuning a menage a trois: Co-evolution and co-adaptation of nuclear and organellar genomes in plants. BioEssays.

[16] Timmis JN, Ayliffe MA, Huang CY, Martin W (2004). Endosymbiotic gene transfer: Organelle genomes forge eukaryotic chromosomes. Nat. Rev. Genet..

[17] O'Conner S, Li L (2020). Mitochondrial fostering: The mitochondrial genome may play a role in plant orphan gene evolution. Front. Plant Sci..

[18] Christensen AC (2013). Plant mitochondrial genome evolution can be explained by DNA repair mechanisms. Genome Biol. Evol..

[19] Best C, Mizrahi R, Ostersetzer-Biran O (2020). Why so complex? The intricacy of genome structure and gene expression, associated with angiosperm mitochondria, may relate to the regulation of embryo quiescence or dormancy-intrinsic blocks to early plant life. Plants.

[20] Yang H (2021). Insights into molecular structure, genome evolution and phylogenetic implication through mitochondrial genome sequence of *Gleditsia sinensis*. Sci. Rep..

[21] Skippington E, Barkman TJ, Rice DW, Palmer JD (2015). Miniaturized mitogenome of the parasitic plant *Viscum scurruloideum* is extremely divergent and dynamic and has lost all *nad* genes. Proc. Natl. Acad. Sci. U. S. A..

[22] Sloan DB (2012). Rapid evolution of enormous, multichromosomal genomes in flowering plant mitochondria with exceptionally high mutation rates. PLoS. Biol..

[23] Sloan DB, Wu Z, Sharbrough J (2018). Correction of persistent errors in *Arabidopsis* reference mitochondrial genomes. Plant Cell.

[24] Goremykin VV, Salamini F, Velasco R, Viola R (2009). Mitochondrial DNA of Vitis vinifera and the issue of rampant horizontal gene transfer. Mol. Biol. Evol..

[25] Raymond O (2018). The Rosa genome provides new insights into the domestication of modern roses. Nat. Genet..

[26] Bi C, Lu N, Xu Y, He C, Lu Z (2020). Characterization and analysis of the mitochondrial genome of common bean (*Phaseolus vulgaris*) by comparative genomic approaches. Int. J. Mol. Sci..

[27] Li H (2021). The complete chloroplast genome sequence of *Photinia × fraseri*, a medicinal plant and phylogenetic analysis. Mitochondr. DNA B.

[28] Aoki K, Matsumura T, Hattori T, Murakami N (2006). Chloroplast DNA phylogeography of *Photinia glabra* (Rosaceae) in Japan. Am. J. Bot..

[29] Small ID, Schallenberg-Rüdinger M, Takenaka M, Mireau H, Ostersetzer-Biran O (2020). Plant organellar RNA editing: What 30 years of research has revealed. Plant J..

[30] Clifton SW (2004). Sequence and comparative analysis of the maize NB mitochondrial genome. Plant. Physiol..

[31] Palmer JD (2000). Dynamic evolution of plant mitochondrial genomes: Mobile genes and introns and highly variable mutation rates. Proc. Natl. Acad. Sci. U. S. A..

[32] Fujii S, Kazama T, Yamada M, Toriyama K (2010). Discovery of global genomic re-organization based on comparison of two newly sequenced rice mitochondrial genomes with cytoplasmic male sterility-related genes. BMC Genom..

[33] Kubo N (2000). Transfer of the mitochondrial rps10 gene to the nucleus in rice: Acquisition of the 5’ untranslated region followed by gene duplication. Mol. Gen. Genet..

[34] Xu Q (2013). The draft genome of sweet orange (Citrus sinensis). Nat. Genet..

[35] Adams KL, Palmer JD (2003). Evolution of mitochondrial gene content: Gene loss and transfer to the nucleus. Mol. Phylogenet. Evol..

[36] Xu Y (2021). Genetic diversity and association analysis among germplasms of *Diospyros kaki* in Zhejiang Province based on SSR markers. Forests.

[37] Tóth G, Gáspári Z, Jurka J (2000). Microsatellites in different eukaryotic genomes: Survey and analysis. Genome Res..

[38] Wee CC (2022). Mitochondrial genome of *Garcinia mangostana* L. variety Mesta. Sci. Rep..

[39] Fang B, Li J, Zhao Q, Liang Y, Yu J (2021). Assembly of the complete mitochondrial genome of Chinese Plum (*Prunus salicina*): Characterization of genome recombination and RNA editing sites. Genes.

[40] Sugiyama Y (2005). The complete nucleotide sequence and multipartite organization of the tobacco mitochondrial genome: Comparative analysis of mitochondrial genomes in higher plants. Mol. Genet. Genom..

[41] Lorizzo M (2012). De novo assembly of the carrot mitochondrial genome using next generation sequencing of whole genomic DNA provides first evidence of DNA transfer into an angiosperm plastid genome. BMC. Plant. Biol..

[42] Gualberto JM (2014). The plant mitochondrial genome: Dynamics and maintenance. Biochimie.

[43] Fischer A, Dotzek J, Walther D, Greiner S (2022). Graph-based models of the Oenothera mitochondrial genome capture the enormous complexity of higher plant mitochondrial DNA organization. NAR. Genom. Bioinform..

[44] Yue J, Lu Q, Ni Y, Chen P, Liu C (2022). Comparative analysis of the plastid and mitochondrial genomes of Artemisia giraldii Pamp. Sci. Rep..

[45] Ichinose M, Sugita M (2016). RNA editing and its molecular mechanism in plant organelles. Genes.

[46] Pinard D, Myburg AA, Mizrachi E (2019). The plastid and mitochondrial genomes of *Eucalyptus grandis*. BMC Genom..

[47] Alverson AJ (2010). Insights into the evolution of mitochondrial genome size from complete sequences of *Citrullus lanatus* and *Cucurbita pepo* (Cucurbitaceae). Mol. Biol. Evol..

[48] Kan SL, Shen TT, Gong P, Ran JH, Wang XQ (2020). The complete mitochondrial genome of *Taxus cuspidata* (Taxaceae): Eight protein-coding genes have transferred to the nuclear genome. BMC. Evol. Biol..

[49] Verhage L (2020). Targeted editing of the *Arabidopsis* mitochondrial genome. Plant. J..

[50] Edera AA, Sanchez-Puerta MV (2021). Computational detection of plant RNA editing events. Methods. Mol. Biol..

[51] Licht K, Jantsch MF (2016). Rapid and dynamic transcriptome regulation by RNA editing and RNA modifications. J. Cell. Biol..

[52] Takenaka M, Zehrmann A, Verbitskiy D, Härtel B, Brennicke A (2013). RNA editing in plants and its evolution. Annu. Rev. Genet..

[53] Cheng Y (2021). Assembly and comparative analysis of the complete mitochondrial genome of *Suaeda glauca*. BMC Genom..

[54] Androsiuk P (2022). Characterization and phylogenetic analysis of the complete mitochondrial genome of the pathogenic fungus Ilyonectria destructans. Sci. Rep..

[55] Hurst LD (2002). The Ka/Ks ratio: Diagnosing the form of sequence evolution. Trends. Genet..

[56] Kozik A (2019). The alternative reality of plant mitochondrial DNA: One ring does not rule them all. PLoS. Genet..

[57] Evans RC, Dickinson TA (1999). Floral ontogeny and morphology in subfamily Amygdaloideae T. & G. (Rosaceae). Int. J. Plant. Sci..

[58] Luan A, Gao A, He J, Bi G, He Y (2017). Characterization of the complete chloroplast genome of black cherry (*Prunus serotina* Ehrh.). Conserv. Genet. Resour..

[59] Robinson JP, Harris SA, Juniper BE (2001). Taxonomy of the genus *Malus* Mill. (Rosaceae) with emphasis on the cultivated apple *Malus domestica* Borkh. Plant. Syst. Evol..

[60] Kalkman C (1973). The malesian species of the subfamily maloideae (Rosaceae). Blumea.

[61] Ruiz C (2018). Diversity of plant defense elicitor peptides within the Rosaceae. BMC. Genet..

[62] Doyle JJ, Doyle JL (1987). A rapid DNA isolation procedure for small quantities of fresh leaf tissue. Phytochem. Bull..

[63] Chen S, Zhou Y, Chen Y, Gu J (2018). fastp: An ultra-fast all-in-one FASTQ preprocessor. Bioinformatics.

[64] Koren S (2017). Canu: Scalable and accurate long-read assembly via adaptive k-mer weighting and repeat separation. Genome. Res..

[65] Hu J, Fan J, Sun Z, Liu S (2020). NextPolish: A fast and efficient genome polishing tool for long-read assembly. Bioinformatics.

[66] Tillich M (2017). GeSeq: Versatile and accurate annotation of organelle genomes. Nucleic Acids. Res..

[67] Chan PP, Lowe TM (2019). tRNAscan-SE: Searching for tRNA genes in genomic sequences. Methods. Mol. Biol..

[68] Lorenz R (2011). ViennaRNA Package 2.0 Algorithms. Mol. Biol..

[69] Mower JP (2005). PREP-Mt: Predictive RNA editor for plant mitochondrial genes. BMC Bioinform..

[70] Stothard P (2000). The sequence manipulation suite: JavaScript programs for analyzing and formatting protein and DNA sequences. Biotechniques.

[71] Sharp PM, Tuohy TM, Mosurski KR (1986). Codon usage in yeast: Cluster analysis clearly differentiates highly and lowly expressed genes. Nucleic. Acids. Res..

[72] Lohse M, Drechsel O, Bock R (2007). OrganellarGenomeDRAW (OGDRAW): A tool for the easy generation of high-quality custom graphical maps of plastid and mitochondrial genomes. Curr. Genet..

[73] Thiel T, Michalek W, Varshney RK, Graner A (2003). Exploiting EST databases for the development and characterization of gene-derived SSR-markers in barley (*Hordeum vulgare* L.). Theor. Appl. Genet..

[74] Wynn EL, Christensen AC (2019). Repeats of unusual size in plant mitochondrial genomes: Identification, incidence and evolution. G3.

[75] Kearse M (2012). Geneious Basic: An integrated and extendable desktop software platform for the organization and analysis of sequence data. Bioinformatics.

[76] Li J (2021). Assembly of the complete mitochondrial genome of an endemic plant, *Scutellaria tsinyunensis*, revealed the existence of two conformations generated by a repeat-mediated recombination. Planta.

[77] Katoh K, Standley DM (2013). MAFFT multiple sequence alignment software version 7: Improvements in performance and usability. Mol. Biol. Evol..

[78] Posada D, Crandall KA (1998). MODELTEST: Testing the model of DNA substitution. Bioinformatics.

[79] Stamatakis A (2014). RAxML version 8: A tool for phylogenetic analysis and post-analysis of large phylogenies. Bioinformatics.

[80] Wang D, Zhang Y, Zhang Z, Zhu J, Yu J (2010). KaKs_Calculator 2.0: A toolkit incorporating gamma-series methods and sliding window strategies. Genom. Proteom. Bioinform..

