# Supplementary material for: Characterization and phylogenetic analysis of the complete mitochondrial genome sequence of Photinia serratifolia
Source: Sci Rep. 2023 Jan 14;13:770. doi: 10.1038/s41598-022-24327-x (PMC9840629; doi:10.1038/s41598-022-24327-x)
Supplement: Supplementary file 1 — Supplementary Information. [file 41598_2022_24327_MOESM1_ESM.zip › Supplemental/Table S3.docx]

**Table S3.** Gene profile and organization of the *P. serratifolia* mitochondrial genome.

| **Group of genes** | **Gene name** | **Length** | **Start codon** | **Stop codon** | **Amino acid** |
| --- | --- | --- | --- | --- | --- |
| ATP synthase | *atp1*(2) | (1533,1277) | ATG | TGA | (511,409) |
|  | *atp4* | 597 | ATG | TAG | 199 |
|  | *atp6* | 990 | ATG | TAG | 330 |
|  | *atp8* | 480 | ATG | TAA | 160 |
|  | *atp9* | 282 | ATG | TAA | 94 |
| Cytohrome c biogenesis | *ccmB* | 621 | ATG | TGA | 207 |
|  | *ccmC* | 753 | ATG | TGA | 251 |
|  | *ccmFC** | 1317 | ATG | TGA | 439 |
|  | *ccmFN* | 1731 | ATG | TAG | 577 |
| Ubichinol cytochrome c reductase | *cob* | 1182 | ATG | TGA | 394 |
| Cytochrome c oxidase | *cox1* | 1488 | ATG | TAA | 496 |
|  | *cox2* | 921 | ATG | TAA | 307 |
|  | *cox3* | 798 | ATG | TGA | 266 |
| Maturases | *matR* | 1968 | ATG | TAG | 656 |
| Transport membrance protein | *mttB* | 720 | TTG | TAG | 240 |
| NADH dehydrogenase | *nad1***** | 888 | ATG | TAA | 295 |
|  | *nad2*** | 918 | ATG | TAA | 306 |
|  | *nad3* | 357 | ATG | TAA | 119 |
|  | *nad4**** | 1488 | ATG | TGA | 496 |
|  | *nad4L* | 303 | ATG | TAA | 101 |
|  | *nad5** | 1437 | ATG | TGA | 479 |
|  | *nad6*(2) | (555,618) | ATG | TAA | (185,206) |
|  | *nad7***** | 1185 | ATG | TAG | 395 |
|  | *nad9* | 573 | ATG | TAA | 191 |
| Ribosomal proteins (LSU) | *rpl15* | 558 | ATG | TAA | 186 |
|  | *rpl10* | 489 | ATG | TAA | 163 |
|  | *rpl16* | 426 | GTG | TAA | 142 |
| Ribosomal proteins (SSU) | *rps1* | 609 | ATG | TAA | 203 |
|  | *rps12* | 378 | ATG | TGA | 126 |
|  | *rps13* | 351 | ATG | TGA | 117 |
|  | *rps14* | 216 | ATG | TAG | 72 |
|  | *rps3* | 1665 | ATG | TGA | 555 |
|  | *rps4* | 1080 | GTG | TAA | 360 |
| Succinate dehydrogenase | *sdh3* | 327 | ATG | TGA | 109 |
|  | *sdh4* | 405 | ATG | TAA | 135 |
| Ribonuclease | rnaseH | 462 | ATG | TAG | 154 |
| Ribosomal RNAs | rrn18 | 1863 |  |  |  |
|  | rrn26 | 3158 |  |  |  |
|  | rrn5(2) | (116,119) |  |  |  |
|  | rrnL | 3349 |  |  |  |
|  | rrnS | 1962 |  |  |  |
| Transfer RNAs | trnM-CAT | 74 |  |  |  |
|  | trnO-TTG | 72 |  |  |  |
|  | trnG-GCC | 72 |  |  |  |
|  | trnfM-CAT(2) | (73,74) |  |  |  |
|  | trnT-TGT | 79 |  |  |  |
|  | trnY-GTA | 83 |  |  |  |
|  | trnN-GTT | 72 |  |  |  |
|  | trnC-GCA | 71 |  |  |  |
|  | trnP-TGG | 75 |  |  |  |
|  | trnF-GAA(2) | (73,74) |  |  |  |
|  | trnS-GCT | 78 |  |  |  |
|  | trnP-GGG | 74 |  |  |  |
|  | trnW-CCA | 74 |  |  |  |
|  | trnS-TGA | 87 |  |  |  |
|  | trnF-GAA | 71 |  |  |  |
|  | trnH-GTG | 74 |  |  |  |
|  | trnE-TTC | 72 |  |  |  |
|  | trnK-TTT | 73 |  |  |  |
|  | trnI-CAT | 77 |  |  |  |
|  | trnD-GTC | 74 |  |  |  |
|  | trnT-TGT | 79 |  |  |  |

Notes: The numbers in bracket after the gene names indicate the duplication number. The number of lowercase * indicates the genes containing introns, and the number of lowercase * indicates the number of introns.
